# Full retraction: El-Remessy AB, Tang Y, Zhu G, Matragoon S, Khalifa Y, Liu EK, Liu JY, Hanson E, Mian S, Fatteh N, Liou GI. Neuroprotective effects of cannabidiol in endotoxin-induced uveitis: critical role of p38 MAPK activation. Mol Vis. 2008; 14:2190-2203.

**Published:** 2014-09-08

The authors made substantive errors in figure images of this article such that the hypotheses were not tested and the conclusions were not supported.

On this basis, the Editors formally retract this article from Molecular Vision.

Sincerely,

The Editors of Molecular Vision

